# Bioactive Compounds in Oxidative Stress-Mediated Diseases: Targeting the NRF2/ARE Signaling Pathway and Epigenetic Regulation

**DOI:** 10.3390/antiox10121859

**Published:** 2021-11-23

**Authors:** Muthu Thiruvengadam, Baskar Venkidasamy, Umadevi Subramanian, Ramkumar Samynathan, Mohammad Ali Shariati, Maksim Rebezov, Shabari Girish, Sivakumar Thangavel, Anand Raj Dhanapal, Natalya Fedoseeva, Joohyun Lee, Ill-Min Chung

**Affiliations:** 1 Department of Crop Science, College of Sanghuh Life Science, Konkuk University, Seoul 05029, Korea; muthu@konkuk.ac.kr; 2 Department of Biotechnology, Sri Shakthi Institute of Engineering and Technology, Coimbatore 641062, Tamil Nadu, India; baskarbt07@gmail.com; 3 Translational Research Platform for Veterinary Biologicals, Tamil Nadu Veterinary and Animal Sciences University, Madhavaram Milk Colony, Chennai 600051, Tamil Nadu, India; umadevi.subramanian@gmail.com; 4 R&D Division Alchem Diagnostics, New No. 1/1, Old No. 78, Gokhale Street, Ramnagar, Coimbatore 641009, Tamil Nadu, India; ramkumarsaha18@gmail.com; 5 Research Department, K.G. Razumovsky Moscow State University of Technologies and Management, 73 Zemlyanoy Val, 109004 Moscow, Russia; shariatymohammadali@gmail.com; 6 School of Agricultural Sciences, Liaocheng University, 34 Wenhua Road, Liaocheng 252000, China; rebezov@ya.ru; 7 Department of Scientific Advisers, V. M. Gorbatov Federal Research Center for Food Systems, 26 Talalikhina Str., 109316 Moscow, Russia; 8 School of Life Sciences, St. Joseph College (Autonomous), Bengaluru 560027, Karnataka, India; shabarisarav@gmail.com; 9 Post Graduate Department of Microbiology, Ayya Nadar Janaki Ammal College, Sivakasi 626124, Tamil Nadu, India; sivasadhana@yahoo.co.in; 10 Department of Biotechnology, Karpagam Academy of Higher Education, Coimbatore 641021, Tamil Nadu, India; rajanandvk88d@gmail.com; 11 Department of Zootechnii, Production and Processing of Livestock Products, Russian State Agrarian Correspondence University, 50 Shosse Entuziastov, 143907 Balashikha, Russia; nfedoseeva0208@yandex.ru

**Keywords:** phytochemicals, NRF2, epigenetic modification, oxidative stress, diabetes, cancer

## Abstract

Oxidative stress is a pathological condition occurring due to an imbalance between the oxidants and antioxidant defense systems in the body. Nuclear factor E2-related factor 2 (NRF2), encoded by the gene *NFE2L2*, is the master regulator of phase II antioxidant enzymes that protect against oxidative stress and inflammation. NRF2/ARE signaling has been considered as a promising target against oxidative stress-mediated diseases like diabetes, fibrosis, neurotoxicity, and cancer. The consumption of dietary phytochemicals acts as an effective modulator of NRF2/ARE in various acute and chronic diseases. In the present review, we discussed the role of NRF2 in diabetes, Alzheimer’s disease (AD), Parkinson’s disease (PD), cancer, and atherosclerosis. Additionally, we discussed the phytochemicals like curcumin, quercetin, resveratrol, epigallocatechin gallate, apigenin, sulforaphane, and ursolic acid that have effectively modified NRF2 signaling and prevented various diseases in both in vitro and in vivo models. Based on the literature, it is clear that dietary phytochemicals can prevent diseases by (1) blocking oxidative stress-inhibiting inflammatory mediators through inhibiting Keap1 or activating *Nrf2* expression and its downstream targets in the nucleus, including HO-1, SOD, and CAT; (2) regulating NRF2 signaling by various kinases like GSK3beta, PI3/AKT, and MAPK; and (3) modifying epigenetic modulation, such as methylation, at the NRF2 promoter region; however, further investigation into other upstream signaling molecules like NRF2 and the effect of phytochemicals on them still need to be investigated in the near future.

## 1. Introduction

Reactive oxygen species (ROS) and reactive nitrogen species (RNS) production are essential for maintaining the body homeostasis and play a key role as regulatory mediators necessary for cell–cell communication or triggering certain signaling pathways. However, at an increased concentration of ROS/RNS in the cellular system, they react with protein, lipids, and DNA molecules and cause detrimental effects in living organisms. In normal conditions, the antioxidant systems, such as catalase, superoxide dismutase (SOD), and glutathione, react with deleterious ROS/RNS into less toxic molecules. During pathological conditions, ROS/RNS overproduction exceeds the antioxidant defense system or dysfunction of antioxidants that are essential to neutralize oxidants, resulting in stress conditions known as oxidative stress. Oxidative stress (OS) will develop into chronic inflammation, leading to various diseases, including diabetes, cardiovascular disease, arthritis, cancer, etc. [1,2].

NRF2 is a redox-sensitive transcription factor encoded by the gene *NFE2L2* that is also the master regulator of phase II antioxidant enzymes that protect against oxidative stress and inflammation. Under normal conditions, NRF2 is sequestered by Kelch-like erythroid cell-derived protein with CNC homology (ECH)-associated protein 1 (KEAP1) as a complex in the cytoplasm where its level is regulated by ubiquitination and proteasomal degradation. Under stress conditions, the interaction between the NRF2 and KEAP1 complexes is broken down, and NRF2 is accumulated in the cytoplasm and then translocated into the nucleus, where it binds to phase 2 of the antioxidant response element (ARE) and initiates the transcription of antioxidative enzymes, such as SOD, catalase, etc. [3,4]. Studies have shown that NRF2 inhibition enhances oxidative stress and inflammation by decreasing phase II antioxidant enzymes that affect the function of the renal system and contribute to the development of hypertension in male C57BL/6J mice [5]. NRF2 activator dimethyl fumarate (DMF) significantly accelerated diabetic wound healing by ameliorating diabetes-mediated oxidative stress in rat macrophage cells incubated with 25-mM glucose [6]. Studies using MCF-7 and SK-BR breast carcinoma cells showed that NRF2 significantly increased the proliferation activity and progression activity of both carcinoma cells and suggested that NRF2 acts as a prognostic factor in breast cancer patients [7].

Phytochemicals such as polyphenols, flavonoids, steroids, organosulfur compounds, and vitamins are plant metabolites that are widely distributed in various parts of plants and essential for plant growth and development. Currently, phytochemicals have been used to prevent and treat various diseases like diabetes, cardiovascular disease, and cancer [8]. Phytochemicals are reported to have antioxidant potential in both in vitro and in vivo studies. Phytochemicals are reported to directly scavenge ROS and enhance the expression of cellular antioxidant enzymes, thus protecting against oxidative stress-mediated cellular injury [9]. Dietary antioxidants have been proven to protect against the progression of diabetes by inhibiting the lipid peroxidation process [10]. The exposure of environmental toxicants to *Drosophila* flies enhanced oxidative stress, leading to neurotoxicity, while the dietary intake of phytochemicals prior to paraquat exposure exhibited neuroprotective and antioxidative potential [11]. Studies have shown that phytochemicals can interact with NRF2 signaling and decrease pathological conditions like neurotoxicity, hepatic injury, etc. The oral administration of curcumin resulted in the accumulation of NRF2 in the cytoplasm and subsequent enhanced nuclear translocation and NRF2, thereby reducing hepatic injury in dimethylnitrosamine (DMN)-induced rats [12]. Naringenin has been well-reported to improve mitochondrial dysfunction and ameliorate oxidative stress in neurons of Sprague–Dawley rats via targeting the NRF2-signaling pathway [13]. In this paper, we will explain the latest update in the research progress on dietary phytochemicals that could effectively target NRF2/ARE signaling under various pathological conditions.

## 2. NRF2-Antioxidant Defense System Pathway

**Figure 1:**
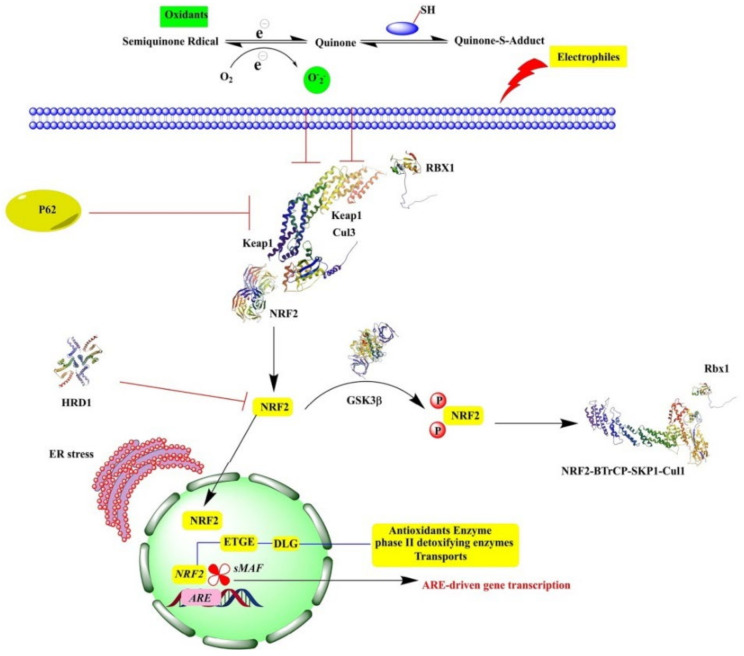
NRF2 activation and nuclear translocation. Cullin3 (Cul3), ring box protein-1 (RBX1), antioxidant response elements (AREs), and the endoplasmic reticulum (ER).

NRF2 is a leucine zipper-type transcription factor belonging to the Cap’n’collar (CNC) family, which is linked with the adaptor protein KEAP1 and remains in the cytoplasm. During unstressed conditions, KEAP1 activates the ubiquitination and proteasomal degradation of NRF2, leading to a lower level of NRF2 in the cytoplasm, which has a short half-life that could last only 20 min under normal conditions. Upon stressful conditions, KEAP1 is exposed to excess ROS and RNS, heavy metals, etc., leading to conformational changes in the structure of KEAP1 and to the release and increased stabilization of NRF2, facilitating the nuclear translocation of NRF2. In the nucleus, it forms a heterodimerization complex with another transcription factor named musculoaponeurotic fibrosarcoma (small Maf). This complex, in turn, binds to ARE in the upstream promoter region and activates the transcription of the target genes, including the antioxidant defense system and phase 2 detoxifying enzymes (Figure 1) [2,14]. Apart from KEAP1, several kinases also target NRF2 expression, including GSK3b, PKC, MAPK, and PERK. Extensive studies on NFE2L2/ARE signaling pathways have shown that NRF2 protects various organs, including the pulmonary, hepatic, neural, and cardiovascular systems. Similar to KEAP1, glycogen synthase kinase (GSK) 3β phosphorylates NRF2 and causes the proteasomal degradation of NRF2. Alternatively, TGF-β-activated kinase (TAK), the mammalian target of rapamycin (mTOR), protein kinase C (PKC), and AMP-activated kinase (AMPK) cause the degradation of Keap1, thereby stabilizing and enhancing NRF2 accumulation in the cytoplasm. NRF2 is positively regulated by three MAP kinases, ERK, JNK, and p38, and the protein kinase (PKR)-like endoplasmic reticulum kinase (PERK) [15]. NRF2/ARE signaling has been considered as a promising target against oxidative stress-mediated diseases like diabetes, fibrosis, neurotoxicity, and cancer [16]. In this review, we will focus on the NRF2/ARE signaling pathway and its role in oxidative stress-mediated diseases.

## 3. NRF2 Signaling in Oxidative Stress-Mediated Diseases

**Figure 2:**
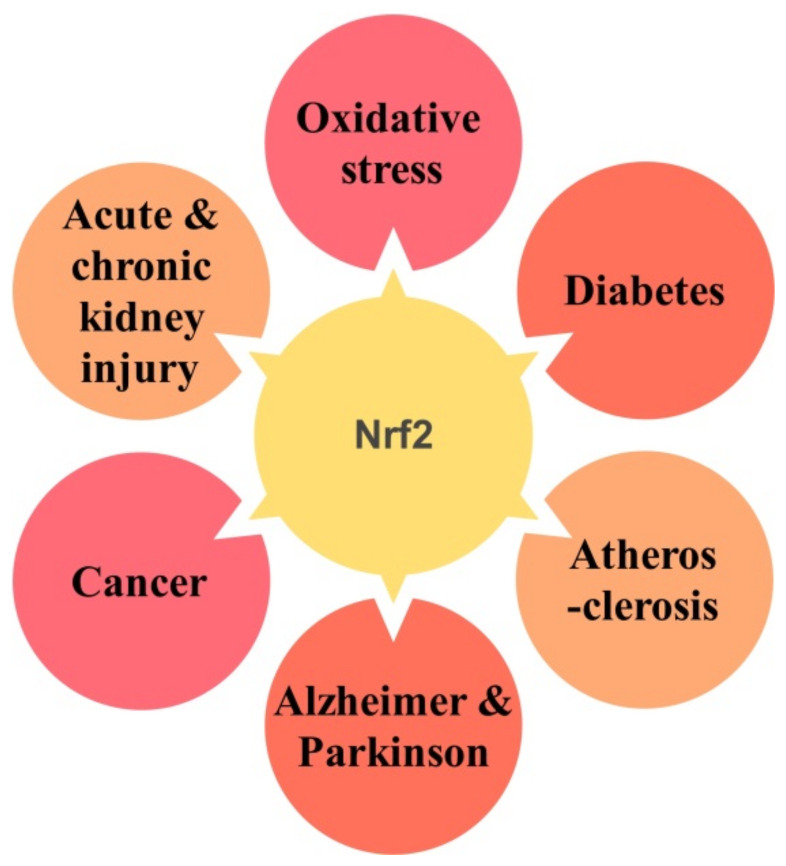
NRF2 signaling pathway is linked with several diseases.

Oxidative stress is a pathological condition occurring due to an imbalance between oxidants and antioxidant defense systems in the body. Oxidative stress is linked with the induction of various diseases, both chronic and acute pathologies, the aging process, and neurodegeneration. In this section, the role of the NRF2 signaling pathway in various diseases, including diabetes, Alzheimer’s disease, kidney injury, and cancer, is discussed (Figure 2).

### 3.1. Diabetes Mellitus

In type 2 diabetes mellitus (T2DM), an increased glycemic overload affects the electron transfer with the mitochondrial membrane, causing an accumulation of free radicals and a depletion of antioxidants, leading to oxidative stress [17]. Oxidative stress is a significant factor in the development of vascular problems in diabetes, most notably T2DM. Insulin resistance is an important feature of T2DM but is also necessary for cardiac complications. Studies have shown that extracellular signal-regulated kinase (ERK) is a negative regulator of glucose uptake and mediates oxidative stress-induced insulin resistance. Additionally, ERK signaling is also involved in the suppression of NRF2 activity in cardiac cells, which is linked to oxidative stress-induced insulin resistance [18]. In another study, it was shown that, due to decreased NRF2/ARE activity, oxidative stress and mitochondrial dysfunctions were enhanced, leading to an insulin resistance and endothelial dysfunction observed in diabetes [19]. Interestingly, studies have shown that, in NRF2 knockout mice, insulin secretion from the pancreatic islets is reduced, whereas NRF2 upregulation eventually increases the insulin-secreting potential of pancreatic β cells [20]. In streptozotocin (STZ)-induced diabetic mice, sulforaphane effectively activated NRF2 expression, leading to the suppression of nephropathy and significantly improving the metabolic parameters such as hyperglycemia, polyuria, polydipsia, and weight loss associated with type II diabetes [21]. In NRF2 knockout mice, the corneal epithelial migration is delayed when compared to that of WT mice, which suggests that NRF2 is mainly responsible for the wound-healing process by promoting cell migration in corneal epithelial cells, and hence, NRF2 might be an attractive therapeutic target of corneal epithelial diseases [22].

### 3.2. Atherosclerosis

Atherosclerosis is a chronic inflammatory disease of large- or medium-sized muscle arteries, while oxidative stress acts mainly as a trigger of atherosclerosis. Due to macrophages’ increased production of ROS, circulating LDL is oxidized, resulting in the formation of foam cells and lipid deposition in the arteries, finally resulting in atherosclerotic plaque [23]. To understand the role of NRF2 in atherogenesis, NRF2-deficient ApoE KO mice (NRF2^−/−^ApoE^−/−^) fed with an atherogenic diet, the data revealed that there was decreased hepatic cholesterol and small atherosclerotic plaques developed in the NRF2^−/−^, ApoE^−/−^ when compared to NRF2^+/+^, ApoE^−/−^ control mice [24]. In low-density lipoprotein (LDL) receptor-deficient mice, NRF2 deficiency in bone marrow-derived cells exacerbates early atherosclerosis. Additionally, the absence of NRF2 results in an increase in the uptake of modified LDL and an increase in the expression of inflammatory markers in thioglycolate-induced peritoneal macrophages, implying that NRF2 has an anti-inflammatory effect in macrophages via increased antioxidant production [25]. In a study, miR-24 expression is decreased in high glucose-induced vascular smooth muscle cells (HG-VSMCs) and, also, in balloon-injured diabetic rats. Upon the deficiency of miR-24, the increased expression of KEAP1 is found together with a decreased expression of NRF2 and heme oxygenase-2 (HO-2) in both HG-VSMCs in diabetic rats. In the same study, the upregulation of miR-24 improved the reendothelialization in balloon-injured diabetic rats, which was due to decreased oxidative stress via the NRF2/HO-1 signaling pathway that restored the activity of antioxidant enzymes SOD and glutathione peroxidize (GPx) in balloon-injured diabetic rats [26].

### 3.3. Alzheimer’s and Parkinson’s Disease

Oxidative stress has been associated with neurological disorders like multiple sclerosis, Alzheimer’s (AD), Parkinson’s disease (PD), etc. AD is mainly due to the abnormal deposition of amyloid β-peptides (Aβ) and accumulation of neurofibrillary tangles containing hyperphosphorylated tau protein and dementia. Studies have shown that oxidative stress increases the aggregation and production of Aβ and, also, stimulates phosphorylation of the tau protein, leading to neurotoxicity. Further, accumulated Aβ and tau protein causes redox imbalance by stimulating dysfunction of the mitochondria, thereby increasing ROS production. Thus, oxidative stress is necessary for the initiation and, also, the progression of AD [27]. Similarly, another study using transgenic mouse models of AD showed that the loss of NRF2 effectively increased the levels of Aβ and phosphorylated tau protein, leading to neurotoxicity [28]. PD is a neurological disorder characterized by the degeneration of dopaminergic neurons in the brain’s substantia nigra. It is considered that oxidative stress is a causal factor in dopaminergic neurotoxicity. Evidence shows that dopaminergic neuronal loss in PD is significantly due to ROS production, which results from low GSH, high levels of iron and calcium, and dopamine metabolism [29]. Skibinski et al. suggested that NRF2 decreases the toxicity induced by α-synuclein and leucine-rich repeat kinase 2 (LRRK2) by maintaining the neuronal protein homeostasis [30]. Additionally, NRF2 is also involved in the clearance of synuclein and converts the aggregation of LRRK2 into inclusion bodies, leading to decreased neuronal toxicity. In a study, acrylamide was exposed to primary astrocytes and microglia obtained from BALB/c mice and evaluated for neurotoxicity. Acrylamide enhanced the ROS and reduced the antioxidant levels, and additionally, the NRF2 and NF-κB pathways were also activated. However, NRF2 activity was increased at the earlier stage of acrylamide exposure, leading to neuronal protection. On the other hand, NF-κB activation occurs at a later stage, leading to the release of proinflammatory mediators like IL-6, TNF-α, and IL-1β, leading to neurotoxicity [31]. Melatonin is a well-known endogenous antioxidant reported to possess neuroprotective effects via the NRF2-signaling pathway and attenuate neuronal apoptosis in HT22 cells [32].

### 3.4. Cancer

**Figure 3:**
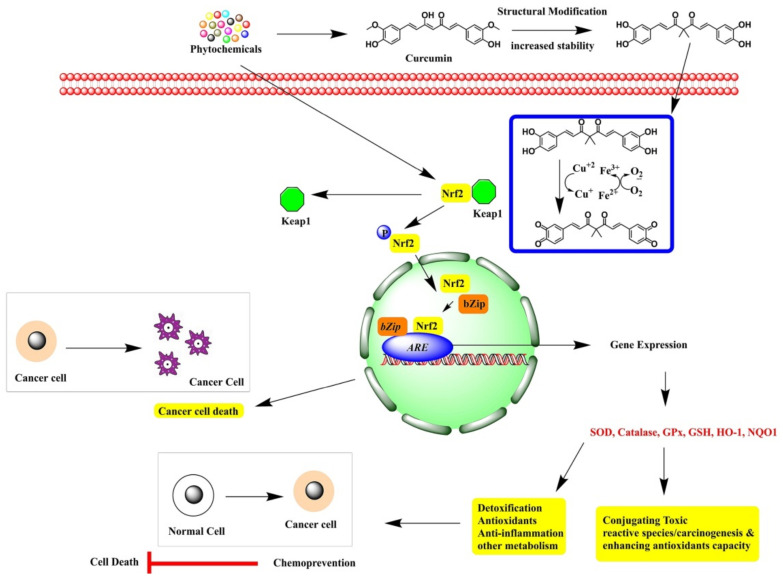
The effects of bioactive compounds in the activation of NRF2 pathways for cancer prevention.

Damage to macromolecules such as protein, DNA, RNA, and membrane lipids occurs as a result of oxidative stress, initiating carcinogenesis. Cancer is a genetic disease in which cells grow out of control and is a multistage process, including initiation, promotion, and progression. Cancer is initiated by ROS-mediated DNA damage, causing gene mutations, genome instability, and altering the DNA structure. In the promotion stage, there will be increased cancer cell proliferation and decreased apoptosis due to abnormal gene expression, improper cell-to-cell communication, and alterations in the second messenger system. In the final stage, oxidative stress involves further DNA alterations and leads to the progression stage of cancer [33]. Additionally, oxidative stress together with inflammatory pathways leads to the transformation of a normal cell into a cancerous cell and further enhances cancer cell survival, proliferation, metastasis, chemoresistance, and radioresistance [33]. NRF2 is a potent transcription activator of genes that are involved in the expression of the antioxidant enzyme system, ubiquitin–proteasome system, anti-inflammatory response, and xenobiotic metabolism. NRF2 activation is beneficial for a host of cells against oxidative stress-related diseases, including cancer. Many cancers exhibit prolonged NRF2 activation due to genetic mutations and promote malignant growth [34]. Accumulating evidence shows that NRF2 signaling pathways are involved in various cancers and are necessary for tumor invasion, metastasis, resistance to apoptosis, and chemoresistance [35]. Activation of the NRF2–ARE pathway is found in the MCF7 breast cancer cell line and is necessary for chemoresistance under hypoxia through the ROS–NRF2–GCLC–GSH pathway [36]. Thus, developing an understanding of the importance of the NRF2–ARE pathway in cancer is an urgent need, and the development of NRF2 inhibitors would provide an opportunity for targeting chemoresistance or development chemotherapy (Figure 3).

### 3.5. Acute and Chronic Kidney Disease

**Figure 4:**
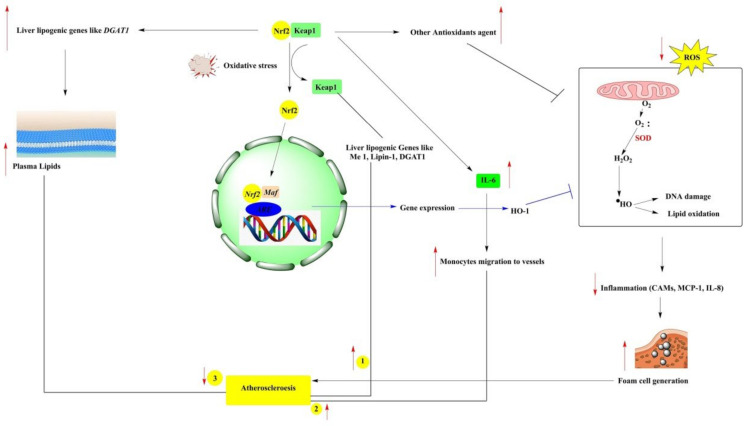
Phytochemicals target NRF2 to inhibit cancer, diabetes, neurotoxicity, cardiac disease, and kidney injury.

Oxidative stress, along with inflammation, is an important factor necessary for the initiation and progression of acute and chronic kidney diseases like diabetic nephropathy, hypertension-associated kidney disease, polycystic disease, and glomerulonephritis. C-reactive protein, IL-6, TNF-α, and fibrinogen, in combination with oxidative stress, induce apoptosis, necrosis, and fibrosis in kidney tissues, eventually leading to chronic kidney disease. Additionally, dysregulated metabolic products like uric acid are also involved in the stimulation of oxidative stress, which would worsen chronic kidney disease [37]. Normally, NRF2 plays a protective role in inducing the expression of antioxidant enzymes and reducing prooxidants. Moreover, studies using the autosomal dominant polycystic kidney disease (ADPKD) mice model showed that the genetic deletion of NRF2 enhanced ROS generation and stimulated cyst growth, and to further confirm that NRF2 was activated using pharmacological inhibitors, the data showed that cyst production and disease progression were slowed down in ADPKD mice [38]. Rush et al. suggested that bardoxolone methyl (BARD) has been used in clinical trials as a pharmacologic NRF2 inducer to treat chronic kidney disease [39]. In another study, Tolvaptan, a vasopressin type 2 receptor antagonist, activated the NRF2/HO-1-signaling pathway through the phosphorylation of PERK in renal cortical collecting duct cell lines and showed that Tolvaptan could be effectively used along with BARD for the treatment of kidney disease [40]. In NRF2-deficient mice when compared to wild-type mice, the severity of acute kidney injury is increased due to decreased renal function, increased expression of tubular injury markers, oxidative stress, endoplasmic reticulum stress, and finally, cell death, suggesting the protective role of NRF2 against kidney injury [41] (Figure 4). 

## 4. Phytochemicals and Regulation of NRF2 Signaling in Various Diseases

**Figure 5:**
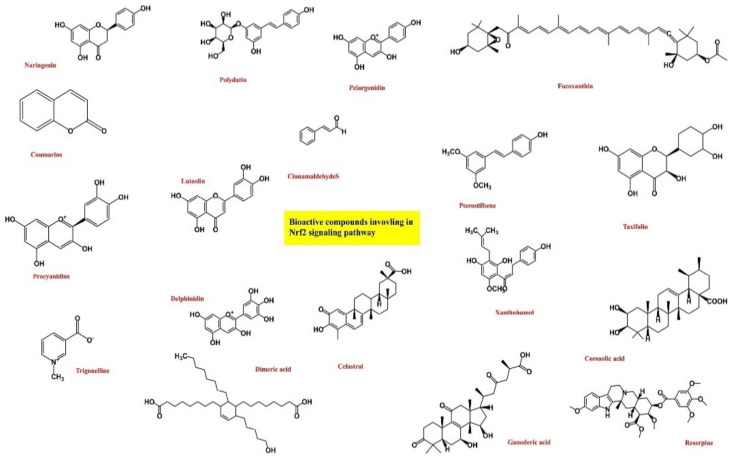
Chemical structure of the bioactive compounds.

Dietary phytochemicals are widely distributed in fruits, grains, herbs, and vegetables and have been considered to possess various biological properties, like antioxidant, anti-inflammatory, anti-cancerous, antifibrosis, etc. Several studies have shown that the consumption of dietary phytochemicals acts as an effective modulator of NRF2/ARE in various acute and chronic diseases. Additionally, phytochemicals effectively regulate the *Nrf2* gene through epigenetic modifications, including histone modifications, miRNA alterations, DNA methylation, etc. In this section, this review provides an update on the role of phytochemicals in regulating NRF2 in various diseases (Figure 4 and Figure 5).

### 4.1. Curcumin

Curcumin is a yellow-colored polyphenol and an active component of the plant *Curcuma longa* (turmeric). It has been known to possess a wide range of biological activities, such as anti-inflammatory, antioxidant, and ameliorating oxidative stress in C2C12 myoblast cells [42]. Curcumin enhances NRF2 expression and stability and promotes the migration of NRF2 to the nucleus, which, in turn, regulates the expression of antioxidant enzyme heme oxygenase-1 (HO-1) and thereby resists oxidative stress and potentially reduces apoptosis in H_2_O_2_-treated RAW264.7 macrophage cells [43]. In a high-fat diet mice model, curcumin attenuated glucose intolerance by decreasing the oxidative stress and improving the nuclear level of NRF2 and its downstream target *HO-1* [44]. Due to the anti-inflammatory potential, the curcumin treatment effectively decreased temporomandibular joint (TMJ) osteoarthritis by inhibiting the expression of inflammatory mediators like IL-6, matrix metalloproteinases (MMPs), a disintegrin, and metalloproteinase with a thrombospondin motif (ADAMTS), a collagen. Additionally, it has been reported that curcumin stimulates the NRF2/ARE-signaling pathway in TMJ-induced inflammatory chondrocytes [45]. Interestingly, curcumin analog A13 also mitigates oxidative stress by activating the NRF2/ARE antioxidant defense pathway and subsequently inhibits fibrosis in the myocardium of both high-fat diet and streptozotocin-induced diabetic rats [46]. Curcumin nanoparticles attenuated Huntington’s disease via the activation of NRF2 and its downstream targets like antioxidant enzymes (superoxide dismutase) and mitochondrial complexes in rats [47]. In another study, the curcumin analog was shown to inhibit prostate cancer by stimulating NRF2 and its downstream genes via decreasing the expression of *Keap1* and, also, decreasing CpG demethylation at the NRF2 promoter by inhibiting DNMT enzymes and HDAC 4 [48]. In rhabdomyolysis-induced acute kidney infection, curcumin, by activating the AMPK, NRF2/HO-1, and PI3K/Akt pathways, effectively inhibited oxidative stress and ameliorated renal injury and apoptosis [49]. Curcumin, at a noncytotoxic concentration, together with cytotoxic 5-fluorouracil, effectively induced apoptosis by decreasing the expression of NRF2 in colorectal cancer cells, which, in turn, suppressed the expression ratio of Bcl-2/Bax and consequently reversed the multidrug resistance of colorectal cancer cells. In cerebral ischemia/reperfusion (I/R) injury, curcumin prevents brain edema and neurological dysfunction by increasing the expression of NRF2 and decreasing the expression of NF-κB through its antioxidant, antiapoptotic potential [50]. Thus, curcumin can be a potential candidate for inducing NRF2 activation and thereby ameliorating various diseases like diabetes, cerebral injury, cancer, and kidney injury.

### 4.2. Quercetin

Quercetin is the most abundant flavonol and is found in onions, berries, apples, cereals, black and green tea, red grapes, broccoli, cherries, etc. Quercetin has beneficial effects in preventing various diseases, such as lung and cardiovascular diseases and cancer, and also acts neuroprotective, antioxidative, antidiabetic, and hepatoprotective [51]. Quercetin, by increasing the NRF2 mRNA expression, increases the antioxidant defense system and reduces the histological abnormalities and thereby protects Doxorubicin-induced cardiomyopathy in rats [52]. In another study, it was shown that quercetin ameliorates oxidative stress-induced ocular disease by upregulating the antioxidant peroxiredoxins by upregulating the NRF2/NRF1 transcription pathway [53]. Quercetin, by inhibiting the ROS/NF-κB/NLRP3 inflammasome/IL-1β and IL-18 pathway and upregulating NRF2 and the anti-inflammatory molecule IL-10, effectively preserves the liver function from alcoholic liver injury [54]. Quercetin exhibited a synergistic effect with sitagliptin and improved cognitive memory by decreasing the Aβ1-42 levels, increasing the expression of NRF2/HO-1, and enhancing the antioxidant activity in the rat brain [55]. Quercetin-conjugated superparamagnetic iron oxide nanoparticles (QCSPIONs) decrease *miR-27a* and increase *Nrf2* and its responsive genes, including *SOD*, *GPX*, and *CAT* in streptozotocin–diabetic rats [56]. In the age-related macular degeneration mice model, the solid dispersion of quercetin significantly reduced the ROS and malondialdehyde content and restored the SOD, catalase, and glutathione peroxidase activities in both the serum and retinal tissues of WT mice when compared to NRF2 KO mice, suggesting that the solid dispersion of quercetin exhibited protective effects by inhibiting retinal oxidative injury through NRF2 activation in mice [57]. In breast cancer cells, quercetin, together with vitamin C, exerted a synergistic effect by decreasing the expression of NRF2 and inducing oxidative stress in the cancer cells. The same study suggested that both compounds can be used as an adjuvant for patients with NRF2-overexpressed cancer [58].

### 4.3. Epigallocatechin-3-Gallate (EGCG) 

Epigallocatechin-3-gallate (EGCG) is the main catechin of green tea and is reported to possess antioxidant, anti-inflammatory effects [59]. In diabetic nephropathy (DN), *Nrf2* expression is enhanced to counteract oxidative stress. However, NRF2 is not translocated into the nucleus, showing a functional impairment of NRF2. Surprisingly, EGCG acts as a booster in translocating NRF2 into the nucleus and activates the NRF2/ARE pathway, which downregulates KEAP1 and its interaction with NRF2, thereby mitigating DN [60]. In another study, EGCG prevents kidney injury by activating NRF2, inhibiting NAPL3 inflammasome and increasing Treg in lupus nephritis-prone mice, suggesting the antioxidant and anti-inflammatory potential of EGCG [59]. The neurological score showed that EGCG exerted neuroprotective effects by improving cerebral functions, attenuating ROS generation, and by activating the NRF2/ARE signaling pathway and its downstream target genes, such as glutamate–cysteine ligase modulatory subunit and glutamate–cysteine ligase regulatory subunit in the middle cerebral artery occlusion rat model of cerebral I/R injury [61]. EGCG exhibits anticancer activity by maintaining an optimum level of NRF2 to overcome the etoposide resistance in lung cancer cells by using various mechanisms: (1) by activating NRF2 in NCIH23 cells and (2) by controlling the *Nrf2* expression via KEAP1-dependent and -independent mechanisms (e.g., the p53/p21 and RAR–RXR pathways) in two adenocarcinoma cells [62]. EGCG effectively inhibited intracellular oxidative stress through the activation of NRF2 signaling and also enhanced the level of the antioxidant enzyme, catalase, in renal tubular cells. The knockdown of NRF2 using siRNA inhibited the protective effects of EGCG, suggesting that EGCG offers protection against oxalate-induced EMT via the NRF2 signaling pathway, and it can be used as an effective treatment strategy against renal fibrosis in the near future [62]. 

### 4.4. Resveratrol

Resveratrol is a natural polyphenolic compound present in berries, grapes, peanuts, and food products such as red wine, and it possesses various biological properties, including antioxidant, anti-inflammatory, and antiplatelet aggregation potential. Resveratrol inhibits diabetes-related cardiac dysfunction and hypertrophy in multiple low doses of streptozotocin-induced diabetic mice by restoring NRF2 and its downstream antioxidative genes, such as *SOD*, *heme oxygenase-1* (*HO-1*), and *metallothionein* (*MT*) [63]. Resveratrol exerts a neuroprotective effect by activating the PI3K/AKT/NRF2 intracellular-signaling pathway and upregulating *HO-1* expression in amyloid-beta Aβ1-42 cytotoxicity-induced PC12 cells [64]. In rotenone-exposed rats, resveratrol downregulated the C/EBP homologous protein (CHOP) and glucose-regulated protein 78, decreased the caspase-3 activity, suppressed the xanthine oxidase activity, and activated the NRF2-signaling pathway in the brain and could thereby be developed as a neuroprotective therapeutic agent for Parkinson’s disease patients [65]. Resveratrol inhibited the proliferation of pancreatic cancer cells and enhanced apoptosis through ROS accumulation. ROS production by resveratrol activated NRF2 and simultaneously inhibited NAF-1. In the same study, it was reported that resveratrol enhanced the sensitivity of gemcitabine via the ROS/NRF2 intracellular-signaling pathway in pancreatic cancer cells [66]. In a mouse model of age-related renal injury, SIRT1/AMPK and PPARα signaling were downregulated, while resveratrol improved the renal function, proteinuria, and glomerulosclerosis by activating NRF2 signaling and activating SIRT1/AMPK and PPARα signaling in the kidney [67]. In high-fat diet (HFD) rats, resveratrol attenuated methylation at the NRF2 promoter region in the liver of mice, which, in turn, reduced lipogenesis by decreasing the expression of genes such as fatty acid synthase and Sterol regulatory element-binding protein (SREBP)-1, suggesting that resveratrol could decrease HG-induced reactive oxygen species production via the NRF2/ARE-signaling pathway [68].

### 4.5. Sulforaphane

Sulforaphane is an isothiocyanate that is found abundantly in cruciferous vegetables such as cabbage, broccoli, and radishes. It showed many biological activities, such as antioxidant, anti-inflammatory, and antitumor activities [69]. In vivo studies using STZ-induced Male Sprague–Dawley rats and in vitro studies using high glucose-induced Müller cells showed that sulforaphane effectively decreased the inflammation and improved the antioxidant enzymes in the injured diabetic retina. Furthermore, sulforaphane inhibited the NLRP3 inflammasome and prevented the keap1-mediated proteasomal degradation of NRF2 and enhanced the stabilization and nuclear accumulation of NRF2 together with increasing the expression of HO-1 and NAD(P)H: quinone oxidoreductase 1 (NQO1), thereby inhibiting diabetic retinopathy [70]. Oxidative stress plays a vital role in the pathogenesis of contrast-induced retinopathy (CIN), while sulforaphane effectively attenuates CIN using both in vitro and in vivo experiments via activating the NRF2 antioxidant defense pathway [71]. β-site APP-cleaving enzyme 1 (BACE1) is the only β-secretase rate-limiting enzyme necessary for the production and accumulation of amyloid-β peptides (Aβ), which is a crucial event involved in the pathogenesis of AD. Studies showed sulforaphane being an effective NRF2 inducer effectively downregulating BACE1 enzymes via upregulating NRF2 target genes, including *HO-1*, and ameliorating cognitive deficits and preventing accumulation in AD-induced mice models [72]. In an in vivo Parkinson’s disease (PD) model using rotenone-mediated neurotoxicity, studies showed that sulforaphane treatment significantly modulated mTOR-mediated p70S6K and 4E-BP1-signaling pathways, inhibited neuronal apoptosis, stimulated the NRF2-dependent reduction of oxidative stress, and ultimately, was involved in the restoration of autophagy, thereby exhibiting the role of sulforaphane as a neuroprotective agent in the PD model [73]. Sulforaphane prevented Ang II-induced cardiomyopathy by activating Akt, which, in turn, inhibits the necessary proteasomal degradation of NRF2. Thus, by activating the AKT/GSK-3β/Fyn pathway, sulforaphane effectively upregulates and activates NRF2 and prevents cardiomyopathy [74].

### 4.6. Apigenin

Apigenin is a naturally occurring plant flavone found richly in fruits and vegetables, including oranges, parsley, celery, etc. It has a variety of biological properties, including the ability to induce apoptosis; prevent DNA damage; and exert immunomodulatory, antioxidant, and anti-inflammatory effects [75]. In high fructose-feeding diet mice, apigenin inhibits the binding of KEAP1 with NRF2, enhances the accumulation and nuclear translocation of NRF2 and its downstream targets *HO-1* and *NQO1*, and ultimately prevents metabolic syndrome [76]. In streptozotocin-induced rats, apigenin–solid lipid nanoparticles effectively prevent diabetic nephropathy by inhibiting the release of inflammatory molecules and reducing the lipid peroxidation process through the NRF2/HO-1/NF-kB-signaling pathway [77]. In LPS-treated BV2 microglial cells, apigenin attenuated TNF-α, IL-1β, and IL-6 production by activating GSK3β/NRF2, suggested to be effective as a therapeutic agent against neurodegenerative diseases [78]. Apigenin acts as a potential chemosensitizer for hepatocellular carcinoma by enabling the sensitization of doxorubicin-treated BEL-7402 cells through the miR101/NRF2-related apoptotic pathway [79]. PI3K/Akt is necessary for glucose uptake and cell survival, and the activation of the PI3K/Akt pathway effectively prevents the development of diabetic neuropathy. In a study, apigenin has been described to activate the PI3K/Akt/NRF2 pathway and improve cell viability in high glucose-treated renal tubular epithelial cells [80]. Apigenin and luteolin at their noncytotoxic concentrations activate the PI3K/NRF2/ARE intracellular pathway system and suppress LPS-induced NO, iNOS, and cPLA2 in human hepatoma HepG2 cells, thereby exerting anti-inflammatory potential [81]. A highly soluble apigenin derivative 6”-O-succinylapigenin showed both neuroprotective and anti-ischemic effects by regulating the ERK/NRF2/HO-1 pathway in a middle cerebral artery occlusion model of male rats [80].

### 4.7. Ursolic Acid

Ursolic acid is a natural pentacyclic triterpenoid acid and is one of the major components present in apples, basil, berries, and fruit peels and is also found in certain medicinal plants. It shows a wide range of biological properties, including antioxidant, anticancer, and anti-inflammatory activities [82]. In middle cerebral artery occlusion mice and NRF2-deficient mice, the inflammatory proteins TLR4 and NF-kB are increased, while the pharmacological activation of NRF2 using ursolic acid effectively decreases the TLR4 and NF-kB pathway in MCAO-treated rats, suggesting the anti-inflammatory role of ursolic acid in cerebral ischemia [83]. Ursolic acid effectively enhanced the expression of Nrf2 via the expression of its upstream factor AKT, while, in the NRF2^(−/−)^ group, ursolic acid inhibited its neuroprotective effect in a traumatic brain injury mice model [84]. In another study, ursolic acid effectively decreased the oxidative stress in osteoblasts via the IER3/Nrf2-signaling pathway [85]. The ursolic acid sensitizing potential is reduced in NRF2 siRNA-transfected HepG2/DDP cells, and the data showed that ursolic acid possesses a sensitizing potential that is mediated via the NRF2/antioxidant response element-signaling pathway [34]. In CCL4-induced liver fibrosis mice, ursolic acid decreased inflammatory markers such as TNF-α, prostaglandin E2, and inducible nitric oxide synthase (iNOS); decreased apoptotic markers like caspase-3; and ultimately, ursolic acid exhibited a hepatoprotective effect against liver fibrosis via the NRF2/ARE-dependent-signaling pathway [86]. In a skin carcinogenesis study, the expression of *Nrf2* and its target gene *HO-1* is reduced in skin tumors. Upon ursolic acid treatment, *Nrf2* expression is restored by decreasing hypermethylated CpG islands of the *Nrf2* gene promoter region in mouse epidermal cells by reducing the expression of epigenetic-modifying enzymes, including the DNA methyltransferases [87].

### 4.8. Naringenin

Naringenin is a bioactive flavonoid found widely in citrus fruits like oranges, grapes, lemons, and tomatoes and is reported to be a well-known antioxidant, anti-inflammatory, and anticancer agent. In a study, naringenin has been reported to possess antidiabetic properties by inhibiting gluconeogenesis, stimulating glycolysis, restoring the insulin levels, and inhibiting apoptosis in both STZ-induced mouse insulinoma cell lines and, also, albino Swiss mice. One of the important mechanisms for the antidiabetic effect of naringenin is by promoting the dissociation of KEAP1 and NRF2 and elevating the antioxidant enzymes like GST in a dose-dependent manner, thereby protecting pancreatic beta cells [88]. Upon exposing mice to an environmental contaminant, perfluorooctane sulfonate, there is an increase in oxidative stress markers like malondialdehyde, hydrogen peroxide, elevated inflammatory mediators like cytokines and interleukins, and stimulated apoptosis, leading to hepatic injury. However, naringenin treatment increased the expression of the NRF2 protein and its target genes *HO1*, *SOD*, and *CAT* and decreased the inflammation and inhibited apoptosis via Bax and caspase-3 in the liver tissue of perfluorooctane sulfonate-exposed mice [89]. Naringenin exerts a neuroprotective effect by mitigating oxidative stress and mitochondrial dysfunction by stimulating the Nrf2-signaling pathway and its target genes *HO-1* and *NQO1* in hypoxia-induced neurons of Sprague–Dawley rats [13]. Controversially, during endometriosis, there is an increased expression of *Nrf2* and its target genes *HO-1* and *NQO1*, together with a decrease in the Keap-1 level, while naringenin effectively modulates the NRF2-mediated-signaling pathway and decreases the invasion of endometrial cells and induces apoptosis [90]. In arsenic-induced rats, *Nrf-2* and mRNA expression are decreased while naringenin targets the NRF2/HO-1 pathway by its antioxidant and anti-inflammatory potential and thereby decreases myocardial injury [91].

### 4.9. Pterostilbene

Pterostilbene is a natural compound and a demethylated analog of resveratrol found in blueberries and grapes. Pterostilbene prevents several diseases, including aging, cardiovascular disease, diabetes, and cancer [92]. Arsenic induces cytotoxicity and stimulates apoptosis in human keratinocyte cells like HaCaT and JB6 by inhibiting the NRF-signaling pathway. Interestingly, pterostilbene enhanced the accumulation of NRF2 in the cytoplasm and subsequently increased the translocation of NRF2 in the nucleus to express its downstream target genes, inhibiting apoptosis and thereby decreasing the skin damage induced by arsenic [93]. In IL-1β-induced chondrocytes, pterostilbene stimulates cytoplasmic stabilization and the nuclear translocation of NRF2 and stimulates its downstream target antioxidant genes and inhibits inflammation, which, altogether, leads to the prevention of chondrocyte damage, cartilage degeneration, and inhibits osteoarthritis [94]. Pterostilbene treatment inhibited gluconeogenesis and stimulated glycolysis in STZ-induced diabetic mice and, also, in MIN6 cells by dissociating the NRF2 and KEAP1 complex and activating NRF2 and enhancing the expression of the downstream target genes of *Nrf2* [95]. Pterostilbene is reported to enhance endoplasmic reticulum stress by causing an imbalance in the redox homeostasis that leads to DNA fragmentation and apoptosis in HeLa cells. In addition, it is reported that the anticancer effect of pterostilbene is mediated by the NRF2-signaling pathway by enhancing the phosphorylation of NRF2, which is associated with the activation of its downstream target genes *GPx*, *GR*, *CAT*, and *NQO1* [96]. In another study, pterostilbene exhibits anticancer effects against cervical cancer by inducing apoptosis, activating the endoplasmic reticulum (ER)/NRF2 pathway, and decreasing the expression of the HPV oncoprotein E6 in cervical cancer cells [97].

### 4.10. Rutin

Rutin is a flavonol predominantly found in buckwheat, mulberry, grapefruit, orange, lemon, and cranberries. Rutin prevents angiogenesis, decreases oxidative stress, decreases cholesterol accumulation, and inhibits inflammation [98]. Rutin potentially upregulated NRF2 and downregulated iNOS in a dose-dependent manner in t-butyl hydroperoxide-induced oxidative stress in human erythrocytes and albino Swiss mice and protects mouse erythrocytes and liver tissue from oxidative stress-mediated toxicity [99]. Rutin, along with ascorbic acid, protects against UV-induced skin damage by effectively decreasing oxidative stress via activating the NRF2/ARE antioxidant pathway in UVB-irradiated human skin fibroblasts [98]. Similarly, rutin in combination with nimesulide was administered for 8 weeks in the STZ model of diabetes in albino Wistar rats. The inflammatory, apoptotic, and oxidative stress markers (caspase 3, TNF-alpha, NF-kB, and MDA) are increased in STZ-induced diabetic rats. Additionally, the NRF2 levels are decreased in diabetic neurons while rutin with nimesulide effectively ameliorates diabetic neuropathy by targeting the NRF-2/HO-1 and NF-kB-signaling pathways [100]. Rutin protects the heart against environmental pollutants such as bisphenol and dibutyl phthalate by decreasing the oxidative stress and inflammation through increasing the expression of NRF2 and decreasing the expression of NF-κB, thus modulating the NRF2/NF-κB-signaling pathway in the hearts of rats, and thus, rutin helps in reducing the risk of developing cardiovascular diseases [101]. Among 19 natural compounds extracted from the *Ginkgo biloba* extracts (GBE), rutin and procyanidin B2 were reported to suppress t-butyl hydroperoxide (t-BHP)-induced oxidative stress in retinal pigment epithelial cells by stimulating *Nrf2* expression together with activating Erk1/2 signaling in injured retinal epithelial cells, thus suggesting that rutin can act as a protective agent in inhibiting oxidative stress-mediated retinal diseases [102]. In another study, brain damage is induced by acrylamide and g-radiation (5Gy), and the protective effect of rutin is analyzed by administering 200-mg/kg/body wt. orally. It was reported that rutin increased the phosphorylation of PI3K, p-AKT, and p-GSK-3β and also upregulated *Nrf2* expression, and it was suggested that the PI3K/AKT/GSK-3β/NRF-2 pathway is involved in the neuroprotective effect of rutin against acrylamide and g-radiation (5Gy)-induced brain damage [103].

### 4.11. Cinnamaldehyde

Cinnamaldehyde, an active component derived from cinnamon, has been described to show diverse biological activities like anticancer, antimicrobial, antidiabetic, and anticoagulant effects [104]. In a study, cinnamon aldehyde inhibited ROS production, decreased type IV collagen and TGF-β1, and thereby ameliorated renal dysfunction and improved endothelium-dependent relaxation of the aorta in diabetic mice. Additionally, cinnamaldehyde enhanced the expression of *Nrf2* and its target genes *HO-1* and *NQO-1* in diabetic mice. However, in *Nrf2*-downregulated mice, the protective effects of cinnamaldehyde decreased, which suggested the crucial role of NRF2 in cinnamaldehyde-treated mice [105]. In LPS-mediated neuroinflammation, trans-cinnamaldehyde showed an anti-inflammatory effect by reducing the IL-1β levels in the hippocampus of mice. Additionally, cinnamaldehyde increased NRF2 nuclear translocation and also increased the nuclear-to-cytoplasmic NRF2 ratio, and ultimately, trans-cinnamaldehyde exhibited antiapoptotic and anti-amyloidogenic effects [104]. In another study, treadmill exercise together with trans-cinnamaldehyde attenuated cognitive dysfunction by decreasing oxidative stress by stimulating the NRF2-signaling pathway and its downstream targets *HO-1*, *NQO-1*, and *SOD* in cognitively impaired mice induced by d-galactose and aluminum chloride [106]. In benzo[a]pyrene-treated HaCaT cells and normal human epidermal keratinocytes, both *Cinnamomum cassia* extract and its active compound cinnamaldehyde activated the NRF2/HO1 pathway and inhibited aryl hydrocarbon receptor signaling, thereby suggested to exhibit a beneficial effect on oxidative stress-mediated diseases [107]. In human hepatocarcinoma (HepG2) cells, cinnamon aldehyde stimulates the ERK1/2, JNK, and AKT pathways without altering the p38 MAPK pathway and ultimately leads to the enhanced nuclear translocation of NRF2 and its downstream target phase II enzyme expression, suggesting the anticancer potential of cinnamaldehyde [108]. Cinnamaldehyde exerts a protective effect against high glucose-induced cardiomyocyte injury by upregulating *Nrf2* and its target genes *HO-1*, *GPx-1*, and *NQO-1* and inhibiting cardiomyocyte hypertrophy in diabetic mice [109]. Upon the cotreatment of quercetin, cinnamaldehyde, and hirudin in high glucose-treated dorsal root ganglion neural cells, there is an increased activation of *Nrf-2/HO-1* and downregulation of the NF-κB pathway and reduced IL-6 and TNF-α levels in neurons suggesting the neuroprotective effect of three phytochemicals [110]. Cinnamaldehyde is reported to increase the *Nrf2* expression and promoted NRF2 nuclear translocation in HUVECs and ultimately suggested that cinnamaldehyde acts as an effective NRF2 activator in high glucose-treated HUVECs [111].

### 4.12. Xanthohumol

Xanthohumol is a significant prenylflavonoid found primarily in hop plants (*Humulus lupulus* L.) and has been shown to have antioxidant and anti-inflammatory properties [112]. In the human pancreatic cancer cell line (PANC-1), the combined treatment of xanthohumol and phenethyl isothiocyanate (PEITC) inhibits the expression of p65 and also decreases the binding efficiency of NF-κB p65 with DNA. Additionally, both compounds effectively activated *Nrf2* expression and its downstream targets *GST*, *NQO1*, and *SOD* and ultimately led to a decreased proliferation of PANC-1 cells, which suggested the anticancer effects of xanthohumol and phenethyl isothiocyanate [113]. In LPS-induced NRF2^−/−^ (knockout) C57BL/6 mice, xanthohumol was reported to suppress acute lung injury via inducing the expression of *Nrf2* through activation of the AMPK/GSK3β pathway and inhibiting the LPS-induced NF-κB-signaling pathway and Txnip/NLRP3 inflammasome [112]. Interestingly, xanthohumol exerts a nephroprotective effect by suppressing the expression of TLR4 and NF-κB via activating the NRF2/HO-1-signaling pathway and suppressing the NF-κB-signaling pathway in a dose-dependent manner during cisplatin-induced nephrotoxicity in C57BL/6 mice [114]. In a study, xanthohumol is reported to exert both chemopreventive effects on normal hepatocytes and be chemotherapeutic in hepatocellular carcinoma cells by activating *Nrf2* and its associated gene expression, including phase II enzymes, in normal cells, while, in cancerous cells, xanthohumol exhibits cytotoxic effects. Additionally, xanthohumol also elevated the expression of *GST*, *HO-1*, *NQO1*, and *P53* in normal hepatocytes [115]. Xanthohumol decreases the inflammatory mediators NO, IL-1β, and TNF-α and prevents the stimulation of NF-κB signaling in LPS-induced microglial BV2 cells. Additionally, xanthohumol stabilized the cytoplasmic level of NRF2 and stimulated the nuclear translocation of NRF2 to activate the intracellular expression of *GSH*, *HO-1*, and *NQO1* in LPS-induced BV2 cells, thereby suggesting the protective role of xanthohumol against brain injury induced by LPS [116]. In another study, it was reported that xanthohumol, through its presence of α,β-unsaturated ketone structure and activating potential of *Nrf2* expression, effectively protects neuronal cells and is suggested as a potential candidate against neurodegenerative diseases [117]. A mechanistic study revealed that xanthohumol exerts a neuroprotective role by binding covalently to the Cys residue(s) in the cytosolic inhibitory protein Keap1 and thereby breaks the KEAP1−NRF2 complex to prevent the proteasome degradation of Nrf2 and, eventually, release NRF2 in PC12 cells [117].

In this review, we also provided additional information on the role of other phytochemicals (Table 1, Table 2 and Table 3) against various diseases targeting NRF2, which showed the importance of NRF2 and, also, to understand the effect of phytochemicals on epigenetic modification. Additionally, Table 2 provides the details of activators and inhibitors of NRF2 that are involved in various diseases. Apart from phytochemicals, the molecules mimicking phytochemicals were also entered into a clinical trial for the treatment of various diseases, including brain ischemia, cancer, etc. To enhance the stability of SFN, Evgen Pharma has developed a cyclodextrin formulation (under the phase II clinical trial), SFX-01, for the treatment of subarachnoid hemorrhage [118]. Yagashita et al. [119] also reviewed that four agents: oltipraz, sulforaphane, dimethyl fumarate, and bardoxolone methyl are in a clinical development that targets NRF2 signaling through interactions with cysteine151 in KEAP1 and suggested that *Nrf2* targeting gene biomarkers was the only biomarker class to be affected positively by all four agents.

## 5. Epigenetic Regulation of Phytochemicals on the NRF2-Signaling Pathway

Sulforaphane promoted demethylation at the NRF2 promoter region, which leads to the activation of *Nrf2* expression in Caco2 cells, suggesting the chemoprevention potential of sulforaphane in colon cancer [139]. Using in vitro and in vivo acute myeloid leukemia (AML) models, researchers discovered that quercetin induces apoptosis by decreasing the nuclear translocation of NRF2 and its proteasomal degradation, as well as stimulating the downregulation of epigenetic-modifying enzymes HDAC and the upregulation of apoptotic-inducing miRNAs, implying that quercetin epigenetically suppresses AML by targeting the NRf2 pathway. In the TPA-induced neoplastic transformation of mouse skin, sulforaphane treatment exhibits the anticancer potential by effectively decreasing the hypermethylation status at the CpG islands of the *Nrf2* gene promoter, leading to increased gene and protein expressions of NRF2 via modulating the protein expression of DNA methyltransferases and histone deacetylases (epigenetic modifying enzymes) [140]. In prostate cancer, NRF2 is epigenetically silenced during the progression of prostate tumorigenesis in mice. However, curcumin administration reversed the methylation status in the CpG promoter region of the *Nrf2* gene, leading to the re-expression of *Nrf2* and its target gene, *NQO-1*, and thereby exerting a chemopreventive effect against prostate cancer [141].

In high glucose-treated HepG2 cells, there is an increased methylation status in the promoter region of NRF2, which could be effectively prevented by resveratrol by inhibiting HG-induced ROS production through demethylation of the NRF2/ARE-signaling pathway [68]. DNA methyltransferase (DNMT) and histone deacetylase (HDAC) assays on mouse skin epidermal JB6 P^+^ cells showed that an apigenin dose dependently prevents skin cancer by effectively reversing the hypermethylated status at the promoter region of the NRF2 promoter, thereby enhancing *Nrf2* expression and the nuclear translocation of Nrf2 in skin epidermal JB6 P^+^ cells [75]. Resveratrol also inhibited 17βestradiol-induced breast carcinogenesis by modulating Nrf2 promoter methylation via miR-93 and stimulating the NRF2/ARE-signaling pathway [142]. γ-tocopherol, a rich mixture of tocopherols, effectively inhibited hypermethylation at the NRF2 promoter in the prostate of transgenic adenocarcinoma of the mouse prostate (TRAMP) cells and mice, leading to the enhanced expression of NRF2, and prevented prostate cancer in the TRAMP model [143]. Luteolin is a dietary flavone reported to induce apoptosis in human colon cancer cells, and additionally, it exhibits demethylation at the NRF2 promoter region and enhances the interaction of NRF2 and p53 that underlies the anticancer effects of luteolin in colon cancer [144]. Natural phytochemicals such as Z-ligustilide and *Radix Angelica Sinensis* prevent DNA hypermethylation at the NRF2 gene promoter region via suppressing DNA methyltransferase activity, which leads to the re-expression of *Nrf2* and its downstream gene targets in TRAMP mice, which underlies the anticancer potential of phytochemicals against prostate cancer [145]. In this section, we have provided details of the phytochemicals that are involved in epigenetic regulation, as shown in Table 3.

**Table 3:** List of phytochemicals modulating the epigenetic changes involved in pathological conditions.

S. No	Phytochemicals	Epigenetic Modification and Mechanism	Cell/Animal Model	Function	Refs
1.	Naringenin	Histone acetylation-dependent inhibition of thioredoxin-interacting protein expression; Regulating AMPK-mediated p300 inactivation	diabetic db/db mouse and INS-1 pancreatic β cell line	Protects pancreatic beta cells and inhibit the progression of type II diabetes	[146]
2.	Pterostilbene	Apoptosis in cancer cells is regulated by theMTA1/HDAC1/NuRD complex	SMMC-7721	suppressed the growth, and invasion of hepatocellular carcinoma	[147]
3.	3,4-dihydroxytoluene, a rutin metabolite	Inhibited p300 histone acetyltransferase activity and induced hypoacetylation at H3K9, H3K36, H4K8 and H4K16. Decreased lipogenesis-related genes and attenuated lipid synthesis	HepG2 cells ob/ob mice	Suppressed the progression of nonalcoholic fatty liver disease	[148]
4.	Cinnamaldehyde	Regulates PERK-CHOP signaling, Inhibits G9a histone methyltransferase, Mediates autophagic cell death	Gastric cancer cells	Induced autophagy-mediated cell death through ER stress and enhanced epigenetic modification in gastric cancer cells	[149]
5.	Xanthohumol	Increased the expression *Nrf2, HMOX1* and *NQO1*	Marc-145 cells.	Reduces PRRSV-induced oxidative stress and inhibits PRRSV growth	[150]
6.	Ganoderic acid	Increased expression of *PKR, PERK, PRDX3, NRF2*	Senescent human amniotic mesenchymal stem cell	Acts as an anti-aging agent	[151]
7.	Celastrol	Deregulation of various miRNA	Hepatocellular carcinoma	Inhibits the progression of hepatocellular carcinoma	[152]
8.	Polydatin	Increases miR200a expression and regulates KEAP1/NRF2 signaling pathway	HepG2 and BRL-3A cells	Reduces fructose-induced liver inflammation and lipid accumulation	[127]
9.	Pelargonidin	inhibits DNA recognition and catalytic binding by DNMT1 and DNMT3A	HT29 cells	Regulates cell cycle and inhibits proliferation of colorectal carcinoma cells	[153]
10.	Delphinidin	Modulate protein expression of DNMT1, DNMT3a, and class I/II HDACs activates the NRF2-ARE pathway	Mouse epidermal JB6 P+ cells	Inhibits neoplastic transformation and acts as an effective skin cancer chemo preventive agent	[129]
11.	Luteolin	regulates NRF2/ARE pathway via modulating DNMTs and HDACs	Human colon cancer cells HCT116	Exerts anti-tumor activity by blocking cell transformation	[154]
12.	Fucoxanthin	Activated NRF2 signaling and reduced DNMT activity	Mouse skin epidermal JB6 P+ cells	Involves in skin cancer prevention and inhibits cell transformation	[155]
13.	Corosolic acid	Modules global CpG methylation at tumor promoter	Mouse epidermal JB6 P+ cells	Acts as an effective agent against skin cancer	[156]

## 6. Conclusions

Oxidative stress and inflammation are the two important factors necessary for the pathogenesis of various diseases, including diabetes, atherosclerosis, kidney injury, AD, PD, cancer, etc. The important mechanism that is necessary for the cellular defense against oxidative stress is the NRF2-signaling pathway. With these concepts, researchers are paying more attention to identifying the agents that could target oxidative stress and inflammation via the NRF2 intracellular-signaling pathway. Among them, phytochemicals are widely used as important therapeutic agents targeting oxidative stress and inflammation. Several lines of evidence support the use of phytochemicals in modulating dysregulated signaling pathways in a variety of disease models. In the present review, we discussed the role of NRF2 during diabetes, AD, PD, cancer, and atherosclerosis. Additionally, we discussed the phytochemicals that have effectively modified NRF2 signaling and prevented various diseases in both in vitro and in vivo models. In this review, we discussed important phytochemicals like curcumin, quercetin, resveratrol, EGCG, apigenin, sulforaphane, and ursolic acid that act as effective NRF2 inducers. Throughout this review, we learned that dietary phytochemicals can prevent diseases by (1) blocking oxidative stress-inhibiting inflammatory mediators through inhibiting Keap1 or activating *Nrf2* expression and its downstream targets in the nucleus, including *HO-1*, *SOD*, and *CAT*; (2) regulating NRF2 signaling by various kinases-like Gsk3beta, PI3/AKT, and MAPK; and (3) altering epigenetic modulation like methylation at the promoter region of NRF2 is also one of the important targets of phytochemicals in preventing disease. Thus, the above three mechanisms can be targeted for oxidative stress-mediated diseases. We have also discussed the miRNA and HDACs that can modify NRF2 in various disease models. However, further investigation into another upstream signaling, histone methylation enzymes targeting NRF2 and the effect of phytochemicals on them still need to be investigated, which might provide additional targets for the prevention of oxidative stress-mediated diseases. This review clearly demonstrates that NRF2/KEAP1 signaling could be an effective target for various pathological conditions, including cancer, Alzheimer’s, etc. Phytochemicals are receiving increased attention due to their role as NRF2 activators and inhibitors; however, much research still needs to investigate the understanding that their pharmacokinetic and dynamic profiles and, also, clinical studies need to be conducted using various phytochemicals and its synthetic moieties against various diseases targeting NRF2 signaling.

## Figures and Tables

**Table 1:** Phytochemicals that target the NRF2-signaling pathway in various diseases.

S.No	Phytochemicals	Molecular Target	Cell/Animal Model	Function	Refs
1.	Luteolin	p62/KEAP1/NRF2	Adult male Sprague−Dawley rats	Neuroprotection	[120]
2.	Fucoxanthin	NRF2 signaling pathway	Skin JB6 P+ cells	Anticancer effect	[76]
3.	Corosolic acid	*Nrf2* expression	Swiss albino mice	Antidiabetic	[121]
4.	Reserpine	Epigenetic modulation of *Nrf2* expression	Skin epidermal JB6 P+ cells	Anticancer	[122]
5.	Taxifolin	NRF2 signaling pathway	Male Swiss Albino Mice	Antioxidant and anti-inflammatory	[123]
6.	Ganoderic acid	*Nrf2* expression	Lung cancer H460 cells	Anticancer	[124]
7.	Celastrol	*Nrf2* expression	Male Wistar albino rat	Antifibrotic	[125]
8.	Polydatin	miR-200a to control KEAP1/NRF2 pathway b	BRL-3A cells	anti-inflammatory and antihyperlipidemic	[126]
9.	Pelargonidin	NRF2 promoter demethylation	Skin epidermal JB6 P+ cells.	Anticancer	[127]
10.	Delphinidin	Epigenetic reactivation of NRF2	Skin epidermal JB6 P+ cells.	Anticancer	[128]

**Table 2:** List of compounds that act as activators and inhibitors of the NRF2-signaling pathway.

S. No	Compounds	Activator/Inhibitor	Cell/Animal Model/	Mechanism	Refs
1.	Ursodiol	FDA approved drug acting as NRF2 activator	KEAP1- knockdown mice, *Nrf2* gene-null mice	Activate NRF2; induces of Mrp family members in livers, stimulates detoxification and antioxidative stress systems	[129]
2.	Dimeric acid	NRF2 activator	Balb/C mice	Activates NRF2, increases hepatic glyoxalase and glutathione, reduces serum and hepatic AGE levels and suppresses inflammatory in MG induced diabetic mice	[130]
3.	Songorine	NRF2 activator	C57BL/6 mice	Activates NRF2/ARE signaling cascades to rescue cardiomyocytes from endotoxin insult and prevents septic heart injury	[131]
4.	Procyanidins	NRF2 inhibitor	A549 cells	Promotes proteasome-independent degradation of nuclear NRF2 via phosphorylating IGF-1 receptor and activating cysteine proteases	[132]
5.	Compound KI-696	KEAP1 Kelch–NRF2 interactions inhibitor	NHBE cells, Bronchial epithelial cells from human COPD patient lung	*Nrf2*-regulated genes are expressed in COPD patient-generated bronchial epithelial cells	[133]
6.	Cyclic peptide head to tail	KEAP1–NRF2-specific protein inhibitor	Mouse RAW 264.7 cells; HepG2-ARE-C8 cells.	Upregulates NRF2-dependent antioxidant proteins and enzymesenhance the antioxidant capacity and inhibit inflammation factors in LPS-induced macrophage RAW 264.7 cells	[134]
7.	Coumarins	Inhibits KEAP1/NRF2 protein–protein interactions	Molecular docking simulation studies	Binds with Keap and activate NRF2 signaling	[135]
8.	Napyradiomycin (Compound **1**)	potent NRF2 activator	BV-2 microglial cells	Exhibits antioxidant and anti-inflammatory effects	[136]
9.	ML385 Small molecule inhibitor	NRF2 inhibitor	Tumor xenograft mice	Improves chemotherapeutic efficacy by interacting with NRF2 and inhibiting its transcriptional activity	[137]
10.	Trigonelline	NRF2 inhibitor	Head and neck cancer cells (HN3R)	Inhibition of the NRF2-ARE mechanism reverses ferroptosis resistance in HNSCC cells	[138]
